# A Screening Tool for Assessing Alcohol Use Risk among Medically Vulnerable Youth

**DOI:** 10.1371/journal.pone.0156240

**Published:** 2016-05-26

**Authors:** Sharon Levy, Fatma Dedeoglu, Jonathan M. Gaffin, Katharine C. Garvey, Elizabeth Harstad, Andrew MacGinnitie, Paul A. Rufo, Qian Huang, Rosemary E. Ziemnik, Lauren E. Wisk, Elissa R. Weitzman

**Affiliations:** 1 Adolescent Substance Abuse Program, Division of Developmental Behavioral Pediatrics, Boston Children’s Hospital, Boston, Massachusetts, United States of America; 2 Department of Pediatrics, Harvard Medical School, Boston, Massachusetts, United States of America; 3 Division of Immunology, Rheumatology Program, Boston Children’s Hospital, Boston, Massachusetts, United States of America; 4 Division of Respiratory Diseases, Boston Children’s Hospital, Boston, Massachusetts, United States of America; 5 Division of Endocrinology, Boston Children’s Hospital, Boston, Massachusetts, United States of America; 6 Division of Developmental Medicine, Boston Children’s Hospital, Boston, Massachusetts, United States of America; 7 Division of Immunology, Boston Children’s Hospital, Boston, Massachusetts, United States of America; 8 Division of Gastroenterology, Boston Children’s Hospital, Boston, Massachusetts, United States of America; 9 Division of Adolescent/Young Adult Medicine, Boston Children’s Hospital, Boston, Massachusetts, United States of America; Penn State College of Medicine, UNITED STATES

## Abstract

**Background:**

In an effort to reduce barriers to screening for alcohol use in pediatric primary care, the National Institute on Alcoholism and Alcohol Abuse (NIAAA) developed a two-question Youth Alcohol Screening Tool derived from population-based survey data. It is unknown whether this screening tool, designed for use with general populations, accurately identifies risk among youth with chronic medical conditions (YCMC). This growing population, which comprises nearly one in four youth in the US, faces a unique constellation of drinking-related risks.

**Method:**

To validate the NIAAA Youth Alcohol Screening Tool in a population of YCMC, we performed a cross-sectional validation study with a sample of 388 youth ages 9–18 years presenting for routine subspecialty care at a large children’s hospital for type 1 diabetes, persistent asthma, cystic fibrosis, inflammatory bowel disease, or juvenile idiopathic arthritis. Participants self-administered the NIAAA Youth Alcohol Screening Tool and the Diagnostic Interview Schedule for Children as a criterion standard measure of alcohol use disorders (AUD). Receiver operating curve analysis was used to determine cut points for identifying youth at moderate and highest risk for an AUD.

**Results:**

Nearly one third of participants (n = 118; 30.4%) reported alcohol use in the past year; 86.4% (106) of past year drinkers did not endorse any AUD criteria, 6.8% (n = 8) of drinkers endorsed a single criterion, and 6.8% of drinkers met criteria for an AUD. Using the NIAAA tool, optimal cut points found to identify youth at moderate and highest risk for an AUD were ≥ 6 and ≥12 drinking days in the past year, respectively.

**Conclusions:**

The NIAAA Youth Alcohol Screening Tool is highly efficient for detecting alcohol use and discriminating disordered use among YCMC. This brief screen appears feasible for use in specialty care to ascertain alcohol-related risk that may impact adversely on health status and disease management.

## Introduction

The developing adolescent brain is particularly vulnerable to harm from alcohol use [1]. Early onset use is associated with a substantially increased lifetime risk of manifesting an alcohol use disorder (AUD). Compared to adults, teens are more susceptible to the most common alcohol-related harms: car crashes and other injuries [2], sexual assault [3], suicide [4], and school failure [5]. To address this burden, the American Academy of Pediatrics recommends that health care providers incorporate screening and brief intervention as a routine component of health supervision to prevent, delay, or reduce alcohol use in children and adolescents [6]. The need for screening and brief intervention to reduce alcohol use has also been recognized globally. The Primary Health Care European Project on Alcohol (PHEPA)’s clinical guidelines address the need for increased education, training, and implementation of screening and brief intervention in primary care settings [7].

One in four US youth and 12% of youth globally are living with a chronic medical condition [8–9]. This medically vulnerable population is growing due to multiple factors: a shift to earlier onset of some chronic conditions [8], rising disease incidence and/or improved survival rates [10–12]. Across conditions, alcohol may play a major unrecognized role in worsening health. In addition to facing the usual risks associated with alcohol use, youth with chronic medical conditions (YCMC) are vulnerable to the potential for alcohol to interact with medications, invalidate lab tests, and lead to co-occurring risk behaviors that adversely affect health. Moreover, alcohol use among YCMC is associated with regular non-adherence to prescription medications [13]. Nonetheless, addressing alcohol use in specialty care settings—the *de facto* medical home for many YCMC—remains exceedingly rare; for example, one previous study found that only 4.2% of adolescents in treatment for rheumatic conditions were screened for alcohol use [14]. Among chronically ill youth, knowledge about alcohol interactions with medications and laboratory testing is generally poor, and poor knowledge is associated with greater risk of alcohol use [13, 15]. Screening and tailored guidance may help to fill knowledge gaps, correct misinformation and ultimately reduce drinking prevalence in this especially vulnerable population.

Asking the ‘right’ questions to help identify adolescent alcohol use and assign level of associated risk for concurrently meeting criteria for an AUD is a critically important driver of early intervention to reduce the global burden of alcohol-related morbidity/mortality [16 –17]. Clinical impressions, which typically rely on the recognition of advanced symptoms for discerning an AUD, have low sensitivity for identifying regular use, early problems, or even a mild to moderate disorder [18]. Validating brief screening tools is a necessary prerequisite to addressing alcohol use and its downstream health consequences.

In 2011, the National Institute on Alcohol Abuse and Alcoholism (NIAAA) produced a two-question Youth Alcohol Screening Tool which asks about the frequency of alcohol consumption and friends’ alcohol use in the past year (Table 1) [19]. The screen’s logic uses frequency of past year alcohol use to triage adolescents into lower, moderate, and highest risk groups with respect to their likelihood of meeting criteria for alcohol dependence based on DSM-IV criteria [20]. The brevity of the tool makes it suitable for broad diffusion including potentially in specialty care clinics or settings where YCMC are seen. We sought to validate the screen’s sensitivity and specificity for assigning risk among youth with chronic medical conditions, updating risk levels based on DSM-5 criteria. Our overarching goal was to facilitate standardized screening of youth with chronic medical conditions using a parsimonious tool that, once validated, could support the response capability of physicians who regularly interact with this group.

**Table 1:** The NIAAA Youth Alcohol Screening Tool. [19]

Age:	First Question:	Second Question:
**Elementary School (ages 9–11)**	**Friends: Any drinking?** “Do you have any friends who drank beer, wine, or any drinking containing alcohol in the ***past year***?”	**Patient: Any drinking?** “How about you—have you ***ever*** had more than a few sips of beer, wine, or any drink containing alcohol?”
**Middle School (ages 11–14)**	**Friends: Any drinking?** “Do you have any friends who drank beer, wine, or any drinking containing alcohol in the ***past year***?”	**Patient: How many days?** “How about you—in the ***past year*, *on how many days*** have you had more than a few sips of beer, wine, or any drink containing alcohol?”
**High School (ages 14–18)**	**Patient: How many days?** “In the ***past year*, *on how many days*** have you had more than a few sips of beer, wine, or any drink containing alcohol?”	**Friends: How much?** “If your friends drink, **how many drinks** do they usually drink on an occasion?”

## Methods

We undertook a cross-sectional screening validation study with a large, medically heterogeneous sample of youth presenting for routine care at subspecialty clinics. Consented youth completed the NIAAA Youth Alcohol Screening Tool and an assessment battery that included criterion standard measures for ascertaining substance use disorder. Both the screen and assessment were administered on a tablet computer with a polarized privacy screen. Parental consent was required for patients aged 9–11 years; we obtained a waiver of parental consent for adolescents ≥ 12 years of age and a federal certificate of confidentiality to protect against disclosure of sensitive information. The study protocol was reviewed and approved by the Boston Children’s Hospital Committee on Clinical Investigations (IRB).

### Participants and Setting

Participants were children aged 9–18 years being treated for Type 1 diabetes (T1D), asthma, cystic fibrosis (CF), inflammatory bowel disease (IBD), or juvenile idiopathic arthritis (JIA) at a large tertiary care children’s hospital. Research assistants screened clinic schedules and approached all age and diagnosis-eligible patients on the day of their visit to invite them to participate. Adolescents that were newly diagnosed (<1 year), medically or emotionally unstable on the day of their appointment, or could not speak or read English or manipulate a tablet computer were excluded. The study and sample were described in detail in a previous publication [21].

### Screening Tool

**Fig 1:**
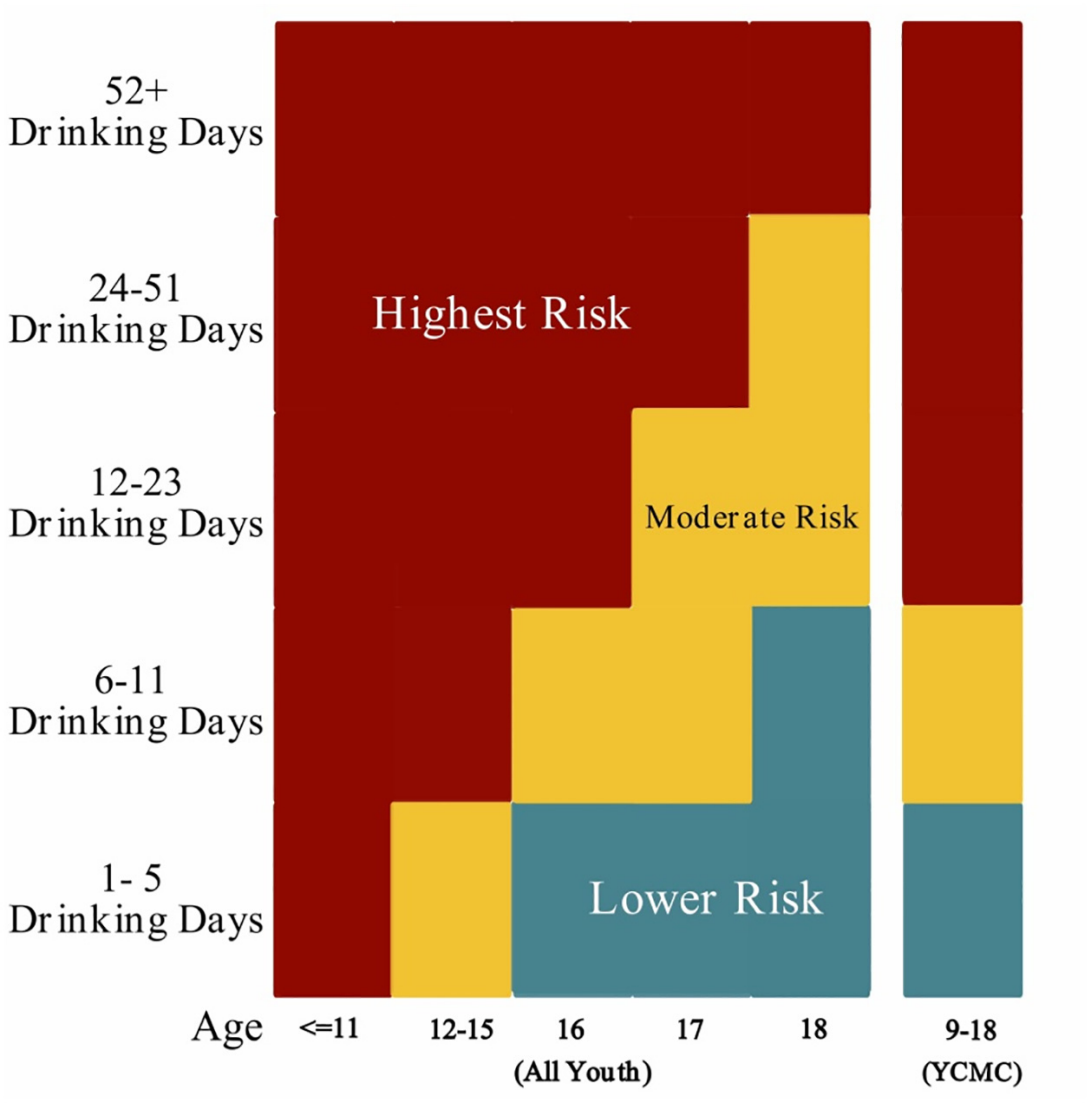
Comparison of estimated risk levels by age and response using NIAAA Youth Alcohol Screening Tool for all youth and YCMC. Screening question assessed the number of drinking days in the past year [19].

The NIAAA Youth Alcohol Screen is comprised of two questions: 1) one about the frequency of past-year alcohol consumption and 2) one about friends’ alcohol use. Minor variations for administration to children and teens of different ages ensure that the tool is developmentally appropriate (Table 1). The number of a subject’s own drinking days in the past year is used to calculate a “risk category” among those who report any past year use (Fig 1). In the original tool risk categories were based on DSM-IV criteria for alcohol abuse and dependence. Participants with past year alcohol use that endorsed any of the DSM-IV criteria for alcohol abuse or alcohol dependence were considered “moderate risk”. Participants with past year alcohol use that endorsed 3 of the 7 DSM-IV criteria for alcohol dependence were categorized as “highest risk” [22]. In DSM-5, the diagnoses of alcohol abuse and dependence were replaced with “alcohol use disorder” which is categorized as mild, moderate or severe. For this analysis we redefined the risk categories by using DSM-5 AUD criteria (see below).

### Assessment Battery

#### Sociodemographics

Respondents reported their age in years, current grade in school, sex (male/female), highest education attained by a parent as a proxy for socioeconomic status (SES) [23], and race/ethnicity (categorized as: non-Hispanic white (only); Hispanic or other race (including: American Indian or Alaska Native, Asian, Black or African American, Native Hawaiian or Other Pacific Islander, White or Other), and unknown.

#### Criterion Standard Measure of Substance Use Disorders

The Diagnostic Interview Schedule for Children (DISC) was self-administered as the criterion standard of past year alcohol and other substance use and alcohol and other substance use disorders [24]. The DISC is comprised of 53 questions that cover all 11 of the DSM-5 criteria for an AUD.

For tobacco and marijuana, we used the initial DISC question “Now think about the last year, have you [smoked cigarettes/snuff/chewing tobacco] or [used marijuana] in the last year?” and for these two substances, we considered an affirmative response as positive for past year use.

Regarding alcohol, participants were categorized into the following four risk groups based on the DISC responses. Subjects that answered “no” to the initial alcohol use DISC question (Now think about the last year. Have you had a drink in the last year?) were considered **Non-users**; those who answered “yes” to the initial DISC question but endorsed 0 of the DSM-5 AUD criteria were classified as **Lower risk**; those who endorsed 1 DSM-5 criterion for AUD were classified as **Moderate risk**; and any diagnosis of AUD (i.e. endorsement of 2 or more DSM-5 AUD criteria) distinguished **Highest risk** [25].

### Data Analysis

All analyses were performed using SAS 9.4 software (SAS Institute, Inc., Cary, North Carolina). We computed descriptive statistics to characterize the study sample in aggregate and by risk categories as defined above; differences in sociodemographic factors across AUD risk categories were evaluated using the Chi-square test.

To assess the validity of the NIAAA Youth Alcohol Screening Tool, we performed three separate receiver operating characteristic (ROC) analyses to estimate the sensitivity and specificity of binary cut-points in number of past year drinking days for predicting lower, moderate, and highest risk of AUD, using macros provided by the SAS Institute [26]. We used each of the cut-points separating the four AUD risk groups to create three binary variables (e.g., one binary variable “lower risk” was created separating categorical AUD risk into no risk vs. ≥ lower risk, while another “moderate risk” was created for <low risk vs. ≥ moderate risk and a final binary variable “highest risk” was created for < moderate risk vs. highest risk). Additionally, we utilized natural cubic splines (S1 File) to test for a nonlinear relationship between drinking days and AUD risk that may have influenced selection of optimal cut-points in number of drinking days for predicting AUD risk, which may approach identified similar cut-points to the standard ROC approach (results available from the authors), hence we present results from the standard ROC curves only.

We estimated odds ratios (ORs) and 95% confidence intervals (CIs) for the associations among friends’ use of alcohol and self-reported substance use using generalized estimating equations controlling for sociodemographics and accounting for clustering within subspecialty clinic (and thus a given condition, e.g., diabetes).

## Results

### Sample characteristics

In total, 388 youth with diabetes, JIA, asthma, cystic fibrosis, and IBD consented to participate (study participation rate 76%). For this study, half (51.5%) of the sample was female and approximately three quarters (75.5%) comprised non-Hispanic white youth.

Overall, nearly one third of participants (30.4%) reported past year alcohol use. Of 118 past year drinkers that completed the DISC, 102 (86.4%) did not endorse any AUD criteria, 8 (6.8%) endorsed a single criterion, and 8 (6.8%) met criteria for an AUD. Older participants and males were more likely to meet criteria for an AUD (p < .05). There were no differences in rates of AUD by race/ethnicity, parental education, or condition (Table 2).

**Table 2:** Sample Sociodemographic Characteristics by Past Year Alcohol Use Disorder (AUD) Risk^a^.

	Total	Past Year Alcohol Use Disorder (AUD) Risk				
	N (%)	Non-users	Lower^b^	Moderate^c^	Highest^d^	p-value^e^
**Total N (%)**	388	270 (69.6)	102 (26.3)	8 (2.1)	8 (2.1)	
Among past year drinkers	118 (30.4)	--	102 (86.4)	8 (6.8)	8 (6.8)	
**Age Groups**						
≤ 11	21 (5.4)	21 (100)	0 (0)	0 (0)	0 (0)	<0.0001
12–15	138 (35.6)	125 (90.6)	13 (9.4)	0 (0)	0 (0)	<0.0001
16	69 (17.8)	56 (81.2)	8 (11.6)	2 (2.9)	3 (4.3)	<0.0001
17	78 (20.1)	34 (43.6)	42 (53.8)	2 (2.6)	0 (0)	<0.0001
18	82 (21.1)	34 (41.5)	39 (47.6)	4 (4.9)	5 (6.1)	<0.0001
**Grade**						
3^rd^- 5^th^	10 (2.6)	10 (100)	0 (0)	0 (0)	0 (0)	0.0001
6^th^%#x2013;8^th^	59 (15.2)	56 (94.9)	3 (5.1)	0 (0)	0 (0)	0.0001
≥ 9^th^	319 (82.2)	204 (63.9)	99 (31.0)	8 (2.5)	8 (2.5)	0.0001
**Sex**						
Male	188 (48.5)	132 (70.2)	43 (22.9)	6 (3.2)	7 (3.7)	0.0324
Female	200 (51.5)	138 (69.0)	59 (29.5)	2 (1.0)	1 (0.5)	0.0324
**Race/Ethnicity**						
Non-Hispanic white	293 (75.5)	201 (68.6)	81 (27.6)	6 (2.0)	5 (1.7)	0.1324
Hispanic or Other race	85 (21.9)	61 (71.8)	21 (24.7)	1 (1.2)	2 (2.4)	0.1324
Unknown	10 (2.6)	8 (80.0)	0 (0)	1 (10.0)	1 (10.0)	0.1324
**Parent’s Education**						
College graduate	271 (69.8)	187 (69.0)	77 (28.4)	5 (1.8)	2 (0.7)	0.1176
Non-college graduate	96 (24.7)	69 (71.9)	20 (20.8)	2 (2.1)	5 (5.2)	0.1176
Don’t know/Unknown	21 (5.4)	14 (66.7)	5 (23.8)	1 (4.8)	1 (4.8)	0.1176
**Chronic Condition**						
Asthma & Cystic fibrosis	97 (25.0)	71 (73.2)	23 (23.7)	2 (2.1)	1 (1.0)	0.7138
Type 1 Diabetes	94 (24.2)	67 (71.3)	21 (22.3)	2 (2.1)	4 (4.3)	0.7138
Inflammatory bowel disease	98 (25.3)	65 (66.3)	28 (28.6)	3 (3.1)	2 (2.0)	0.7138
Juvenile idiopathic arthritis	99 (25.5)	67 (67.7)	30 (30.3)	1 (1.0)	1 (1.0)	0.7138

^a^ Data are presented as number (percentage) of participants; the ‘Total’ column provides column percentages while row percentages are included for AUD risk.

^b^ Lower risk: past year alcohol use, 0 DSM-5 Alcohol Use Disorder (AUD) criteria

^c^ Moderate risk: past year alcohol use and endorsement of 1 DSM-5 criterion for AUD

^d^ Highest risk: any diagnosis of AUD (i.e. endorsement of 2 or more DSM-5 AUD criteria)

^e^ Chi-square test for difference across categories.

### Concurrent Validity of NIAAA Youth Alcohol Screening Tool

For our sample, 70.4% of youth reported no past year drinking days, 16.8% reported between 1–5 days, 5.4% reported between 6–12 days and 7.5% reported ≥13 days. ROC curves showed that the optimal cut points to identify youth at moderate and highest risk for an AUD disorder were ≥ 6 (sensitivity: 1.00, specificity: 0.91 (95%CI: 0.88–0.94)) and ≥13 (sensitivity: 1.00, specificity: 0.94 (95%CI: 0.92–0.97)) drinking days in the past year, respectively (Table 3).

**Table 3:** Cut-points in drinking days used to identify AUD risk: Prevalence, Sensitivity, Specificity, and Area Under the ROC Curve.

AUD Risk Categorization	Cut-points (drinking days)	% at/above cut-point	Sensitivity (95% CI)	Specificity (95% CI)	AUROC^d^
**Lower**^a^	0 vs. ≥1	29.6%	0.83 (0.76–0.90)	0.94 (0.91–0.97)	0.903
**Moderate**^b^	≤5 vs. ≥6	12.9%	1.00 (--)	0.91 (0.88–0.94)	0.962
**Highest**^c^	≤12 vs. ≥13	7.5%	1.00 (--)	0.94 (0.92–0.97)	0.980

^a^ Lower risk: Any past-year alcohol use, but no DSM-5 Alcohol Use Disorder (AUD) criteria

^b^ Moderate risk: Any past year alcohol use and endorsement of 1 DSM-5 criterion for AUD

^c^ Highest risk: Any diagnosis of AUD (i.e. endorsement of 2 or more DSM-5 AUD criteria)

^d^ AUROC: Area Under ROC (Receiver Operating Characteristic) Curve

### Friends’ Use (NIAAA Youth Alcohol Screening Tool)

Older age was associated with reporting friends’ alcohol use on the NIAAA tool (p <.0001). None of the participants in elementary school, and only 7.0% of youth in middle school (grades 6–8) reported having friends who drank in the past year. In contrast, more than one half (59.2%) of all youth in high school reported that their friends drink, and one in five (20.3%) reported that their friends binge drink. There was no association between friends’ use and the respondent’s medical condition, gender, race/ethnicity, or parent’s education (S1 Table). High school youth who reported that their friends drink had significantly higher odds of reporting past year drinking themselves (adjusted OR 12.60; 95% CI 4.82–32.89). High school youth who reported that their friends binge drink had higher odds of any substance use (including alcohol, marijuana or tobacco, OR: 16.45, 95% CI: 5.35–50.64) and meeting criteria for a substance use disorder (OR: 43.63, 95% CI: 6.63–286.98) compared to those who reported their friends do not drink at all (S2 Table).

## Discussion

Among a large, medically heterogeneous sample of youth being treated for a chronic disease, we found that nearly one third (30.4%) report past year alcohol consumption and 6.8% of drinkers met DSM-5 criteria for an AUD. Although this rate did not vary across medical conditions, males were more likely to meet criteria for an AUD, similar to data reported in previous studies [27–28].

In our study, a report of 6 or more drinking days during the past year had excellent sensitivity/specificity for detecting moderate risk and a report of 13 or more drinking days (about once a month) was highly sensitive/specific for detecting an AUD. Information such as this may be easy to obtain and highly informative for guiding brief interventions for this population of youth. When measured against a lengthy diagnostic standard, the brief frequency-based question approach of the Youth Alcohol Screening Tool appears to offer a parsimonious and informative strategy for ascertaining alcohol use risk for youth with chronic medical conditions. While sample size for this project prohibited us from examining single year age groups, we found that frequency cut-offs were similar to the NIAAA empirically derived cut-offs. In our analysis, 13 or more past year drinking days denoted highest risk which matched the NIAAA highest risk cut-off for 12–16 year olds; 6 or more drinks denoted moderate risk which matched the NIAAA moderate risk cut-off for 16 and 17 year olds. Findings are similar to the “Screening To Brief Intervention” (S2BI) screening tool [29], that uses a past-year frequency question (“In the past year, how many times have you consumed a drink with alcohol in it?”) with forced choice responses (never, once or twice, monthly, weekly or more) to assess risk of AUD; monthly drinking was found to be sensitive and specific for identifying mild to moderate AUDs among adolescents 12–17 years of age. Findings indicate notably higher frequency thresholds than those reported by Kelly et al (2014), who validated an identically phrased past year frequency question against a different criterion standard measure for substance use disorders (modified CIDI-SAM) and found that >1 day of past year alcohol use was sensitive and specific for identifying AUDs in a population of primarily African American, inner city general pediatric patients ages 12 to 18 [30]. Discrepancies suggest that youth alcohol screening tools may operate differently across population subgroups, requiring further calibration. Consistent with the original description of the NIAAA Youth Alcohol Screening Tool [19], we found that friends’ alcohol use was highly correlated with both self-reported drinking and AUDs.

Accurate assessment of risk level is critical to guiding an appropriate response from a clinician that engages patients in a dialogue about preventing or reducing alcohol use. For non-users and “lower risk” users, for whom problems associated with use are unlikely and thus ambivalence regarding use may be minimal, physicians are advised to give a prevention or cessation message and accompanying brief medical advice on the harmful effects of alcohol use [6]. For those in the moderate and highest risk groups for whom problems associated with use are more likely, physicians are advised to use brief interventions (BI) targeting behavior change, which have been shown to reduce alcohol use, [31–34] and associated problems [33–35] and to be cost-effective compared with brief education [36]. Several BI models have been evaluated in primary care and found to be modestly successful in demonstrating reductions in substance use and related consequences and/or risky behaviors, including: the structured “5A’s” intervention “CHAT,” with follow-up “Healthy Choices,” and “MOMENT” [37–40]. In one study in which physicians were trained to deliver the “5 A’s” intervention, a structured brief intervention that uses the principles of motivational interviewing, to patients along with usual care, physicians’ clinical instincts without the aid of a screening tool had poor sensitivity for identifying highest risk patients; only one-third of binge drinkers were identified and received the intervention, further underscoring the need for screening with a validated tool in order to guide administration of the appropriate intervention [37–41].

This validation study establishes that the risk of an alcohol disorder in a population of youth with chronic medical conditions is associated with the same drinking day frequency as that found among youth in general; this is important knowledge for guiding clinical intervention efforts. Nevertheless, it may be that the greatest alcohol-related risk faced by these youth occurs in the context of non-adherence to prescription medications. Past-year alcohol consumption by YCMC is associated with a nearly 80% increase in risk for regular medication non-adherence [21], an important intermediary marker of poor outcomes for medically vulnerable youth [42]. Therefore, even “lower risk” alcohol use, which may be missed entirely without the aid of universally administered standardized screening tools, may pose a serious threat to the health of this group.

### Limitations

This is the first study to validate use of a brief screening tool to detect AUD patterns among youth with chronic medical conditions, and the data should be viewed within the context of several limitations. We validated the NIAAA Youth Alcohol Screening Tool in a medically heterogeneous population of males and females of mixed race and socioeconomic status obtaining care at a single medical institution. Results may not be generalizable to youth with chronic medical conditions treated at other centers, or in a general, rather than a specialty, pediatric practice. Few participants in our sample endorsed any of the AUD criteria. In our previous work conducted in a primary care setting with children and adolescents aged 12–17 years, we found 13.6% met criteria for an AUD [43], greater than the 6.8% in this report. We note that this study included children as young as 9 which likely contributes to difference noted. Despite the overall small number of participants who met criteria for an AUD, ROC curves indicated a high degree of precision in risk estimation. Our sample included a small number of children under the age of 12, and none of these children reported past year alcohol use, hence we are unable to comment on the properties of the tool for children in this age group. This cross sectional validation of a screening tool was not intended to predict *future* risk of AUD, and clinicians who take care of children and adolescents should be cognizant of the risk of early initiation of *any* alcohol use, regardless of whether AUD criteria are met. Finally, our data relied on self-reported substance use which has the potential for minimization or exaggeration, though despite these limitations remains the criterion standard for this type of research and has been shown to accurately reflect these behaviors [44]. To support honest reporting, we explained to participants that their answers would be kept confidential (not revealed to parents or providers) with exceptions only if a response suggested that a participant was at elevated risk of acute harm. Research with adolescents has shown that offering ‘partial confidentiality’ such as this is positively correlated with willingness to divulge sensitive information [45].

## Conclusion

Among youth with chronic medical conditions, alcohol use is common. Detecting disordered use among medically vulnerable youth is highly efficient using the NIAAA Youth Alcohol Screening Tool. Incorporation of validated alcohol screening tools into subspecialty as well as primary medical care constitutes an important step toward addressing the potential for negative impacts on health from alcohol use among the large and growing population of chronically ill youth. Future research is needed to identify salient prevention messages that can reach chronically ill youth as is rigorous evaluation of the effects on drinking behaviors and health outcomes of tailored/targeted screening, brief intervention and referral to treatment programs.

## Supporting Information

S1 File Use of natural cubic splines to evaluate a nonlinear relationship between drinking days and alcohol use disorder risk. (DOCX)

S1 Table Associations of sociodemographic characteristics with friends’ past year alcohol use among high school youth. (DOCX)

S2 Table Multivariate logistic regression predicting high school youth’s own risk for past year use of substance as predicted by friends’ past year use. (DOCX)
