# Supplementary material for: A Screening Tool for Assessing Alcohol Use Risk among Medically Vulnerable Youth
Source: PLoS One. 2016 May 26;11(5):e0156240. doi: 10.1371/journal.pone.0156240 (PMC4882018; doi:10.1371/journal.pone.0156240)
Supplement: S1 File — (DOCX) [file pone.0156240.s001.docx]

**Supporting Figure 1. Use of natural cubic splines to evaluate a nonlinear relationship between drinking days and alcohol use disorder risk.**

Family: gaussian

Link function: identity

Formula: risk ~ s(alcdays)

Parametric coefficients:

Estimate Std. Error t value Pr(>|t|)

(Intercept) 0.36598 0.01851 19.77 <2e-16 ***

---

Approximate significance of smooth terms:

edf Ref.df F p-value

s(alcdays) 8.113 8.754 88.6 <2e-16 ***

---

Signif. codes: 0 ‘***’ 0.001 ‘**’ 0.01 ‘*’ 0.05 ‘.’ 0.1 ‘ ’ 1

R-sq.(adj) = 0.666 Deviance explained = 67.3%

GCV = 0.13612 Scale est. = 0.13293 n = 388


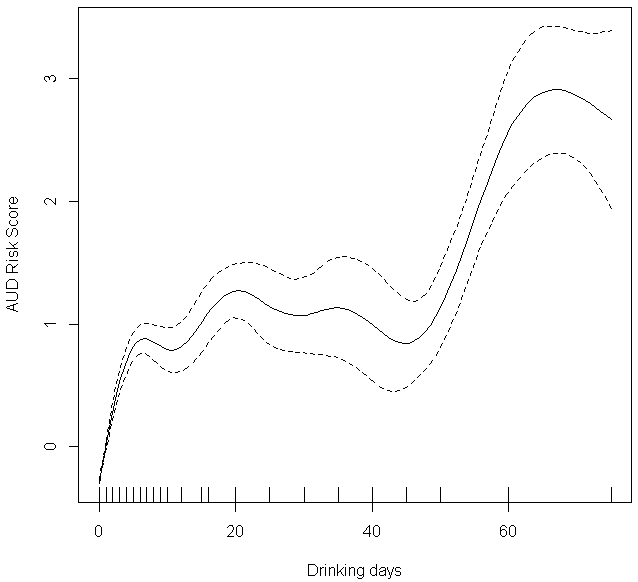


Family: poisson

Link function: log

Formula: risk ~ s(alcdays)

Parametric coefficients:

Estimate Std. Error z value Pr(>|z|)

(Intercept) -1.6274 0.1302 -12.5 <2e-16 ***

---

Approximate significance of smooth terms:

edf Ref.df Chi.sq p-value

s(alcdays) 8.046 8.712 174.4 <2e-16 ***

---

Signif. codes: 0 ‘***’ 0.001 ‘**’ 0.01 ‘*’ 0.05 ‘.’ 0.1 ‘ ’ 1

R-sq.(adj) = 0.624 Deviance explained = 55.3%

UBRE = -0.53823 Scale est. = 1 n = 388


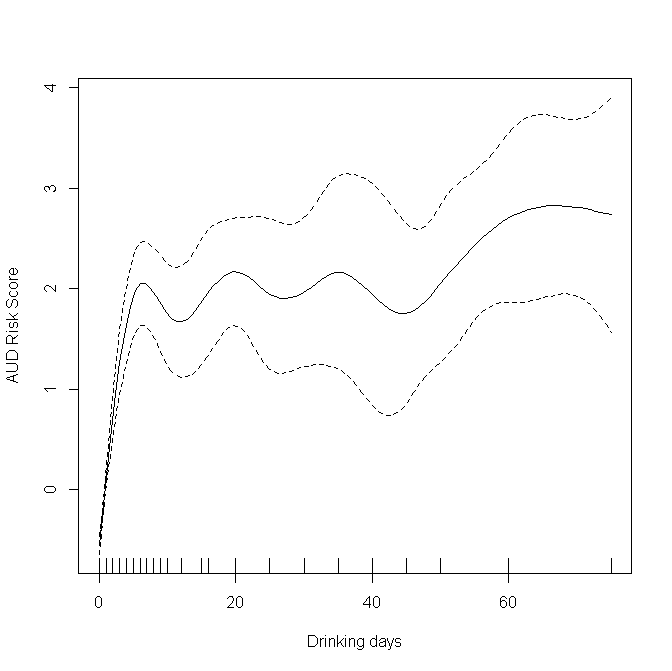


Family: gaussian

Link function: identity

Formula: risk ~ s(alcdays) + s(age)

Parametric coefficients:

Estimate Std. Error t value Pr(>|t|)

(Intercept) -0.121391 0.152821 -0.794 0.42750

age 0.031181 0.009707 3.212 0.00143 **

---

Approximate significance of smooth terms:

edf Ref.df F p-value

s(alcdays) 8.061 8.728 70.84 <2e-16 ***

---

Signif. codes: 0 ‘***’ 0.001 ‘**’ 0.01 ‘*’ 0.05 ‘.’ 0.1 ‘ ’ 1

R-sq.(adj) = 0.673 Deviance explained = 68.1%

GCV = 0.13341 Scale est. = 0.12995 n = 388


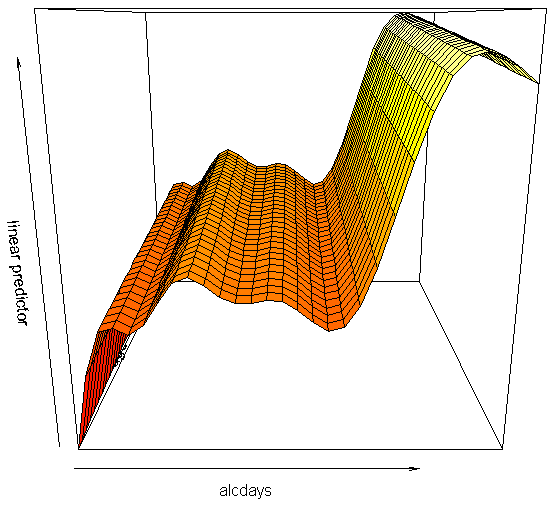


Family: poisson

Link function: log

Formula: risk ~ s(alcdays) + s(age)

Parametric coefficients:

Estimate Std. Error z value Pr(>|z|)

(Intercept) -5.64409 1.25657 -4.492 7.07e-06 ***

age 0.25031 0.07619 3.285 0.00102 **

---

Approximate significance of smooth terms:

edf Ref.df Chi.sq p-value

s(alcdays) 7.865 8.604 109.4 <2e-16 ***

---

Signif. codes: 0 ‘***’ 0.001 ‘**’ 0.01 ‘*’ 0.05 ‘.’ 0.1 ‘ ’ 1

R-sq.(adj) = 0.619 Deviance explained = 58.3%

UBRE = -0.56202 Scale est. = 1 n = 388


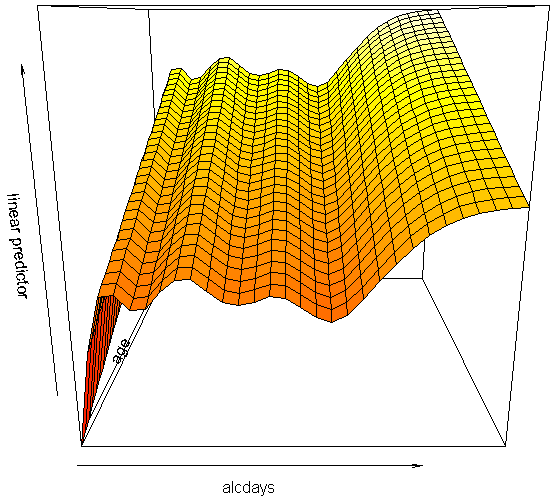


From the above spline models, we identified inflection points at 3 drinking days and 16 drinking days. The cut-points/risk groups are as follows:

No risk 0 drinking days N=273

Low risk 1-3 drinking days N=50

Moderate risk 4-16 drinking days N=40

High risk 17+ drinking days N=25

*Note that these groups are highly correlated (pearson=0.971, spearman=0.996) to the ROC groupings.

The following models assess the association of these cut-points/ risk groups as predictors of AUD risk.

**UNADJUSTED -** Call: glm(formula = risk ~ as.factor(level2), family = gaussian(), data = niaaa)

Deviance Residuals: Min=-1.15000 Max=1.85000

Coefficients:

Estimate Std. Error t value Pr(>|t|)

(Intercept) 0.07326 0.02351 3.116 0.00197 **

as.factor(level2)1 0.60674 0.05976 10.153 < 2e-16 ***

as.factor(level2)2 1.07674 0.06577 16.371 < 2e-16 ***

as.factor(level2)3 1.60674 0.08118 19.793 < 2e-16 ***

---

Signif. codes: 0 ‘***’ 0.001 ‘**’ 0.01 ‘*’ 0.05 ‘.’ 0.1 ‘ ’ 1

(Dispersion parameter 0.150924)

Null deviance: 154.031 on 387 degrees of freedom

Residual deviance: 57.955 on 384 degrees of freedom

AIC: 373.38

**ADJUSTED -** Call: glm(formula = risk ~ as.factor(level2) + age + as.factor(sex) + as.factor(white) + as.factor(college), family = gaussian(), data = niaaa)

Deviance Residuals: Min=-1.08871 Max=1.60446

Coefficients:

Estimate Std. Error t value Pr(>|t|)

(Intercept) -0.266796 0.169255 -1.576 0.1158

as.factor(level2)1 0.576925 0.062075 9.294 <2e-16 ***

as.factor(level2)2 1.025112 0.068396 14.988 <2e-16 ***

as.factor(level2)3 1.560775 0.085495 18.256 <2e-16 ***

age 0.024629 0.010552 2.334 0.0201 *

as.factor(sex)2 -0.008381 0.039747 -0.211 0.8331

as.factor(white)1 -0.015369 0.049631 -0.310 0.7570

as.factor(white)2 -0.003031 0.158839 -0.019 0.9848

as.factor(college)1 -0.039914 0.047877 -0.834 0.4050

as.factor(college)2 0.205317 0.113526 1.809 0.0713 .

---

Signif. codes: 0 ‘***’ 0.001 ‘**’ 0.01 ‘*’ 0.05 ‘.’ 0.1 ‘ ’ 1

(Dispersion parameter 0.1477763)

Null deviance: 154.031 on 387 degrees of freedom

Residual deviance: 55.859 on 378 degrees of freedom

AIC: 371.09

The ROC curve analysis identified inflection points at 5 drinking days and 12 drinking days. The cut-points/risk groups are as follows:

No risk 0 drinking days N=273

Low risk 1-5 drinking days N=65

Moderate risk 6-12 drinking days N=21

High risk 13+ drinking days N=29

The following models assess the association of these cut-points/ risk groups as predictors of AUD risk.

**UNADJUSTED -** Call: glm(formula = risk ~ as.factor(level1), family = gaussian(), data = niaaa)

Deviance Residuals: Min=-1.19048 Max=1.34483

Coefficients:

Estimate Std. Error t value Pr(>|t|)

(Intercept) 0.07326 0.02360 3.104 0.00205 **

as.factor(level1)1 0.68059 0.05382 12.647 < 2e-16 ***

as.factor(level1)2 1.11722 0.08830 12.652 < 2e-16 ***

as.factor(level1)3 1.58191 0.07616 20.772 < 2e-16 ***

---

Signif. codes: 0 ‘***’ 0.001 ‘**’ 0.01 ‘*’ 0.05 ‘.’ 0.1 ‘ ’ 1

(Dispersion parameter 0.1520473)

Null deviance: 154.031 on 387 degrees of freedom

Residual deviance: 58.386 on 384 degrees of freedom

AIC: 376.25

**ADJUSTED -** Call: glm(formula = risk ~ as.factor(level1) + age + as.factor(sex) + as.factor(white) + as.factor(college), family = gaussian(), data = niaaa)

Deviance Residuals: Min=-1.12947 Max=1.39375

Coefficients:

Estimate Std. Error t value Pr(>|t|)

(Intercept) -0.28792 0.16988 -1.695 0.0909 .

as.factor(level1)1 0.64392 0.05666 11.365 <2e-16 ***

as.factor(level1)2 1.06297 0.09056 11.738 <2e-16 ***

as.factor(level1)3 1.52963 0.08038 19.029 <2e-16 ***

age 0.02589 0.01059 2.444 0.0150 *

as.factor(sex)2 -0.01012 0.03999 -0.253 0.8004

as.factor(white)1 -0.01789 0.04988 -0.359 0.7201

as.factor(white)2 -0.07715 0.15986 -0.483 0.6297

as.factor(college)1 -0.03174 0.04818 -0.659 0.5104

as.factor(college)2 0.23006 0.11411 2.016 0.0445 *

---

Signif. codes: 0 ‘***’ 0.001 ‘**’ 0.01 ‘*’ 0.05 ‘.’ 0.1 ‘ ’ 1

(Dispersion parameter 0.1490541)

Null deviance: 154.031 on 387 degrees of freedom

Residual deviance: 56.342 on 378 degrees of freedom

AIC: 374.43
