# Supplementary material for: A Screening Tool for Assessing Alcohol Use Risk among Medically Vulnerable Youth
Source: PLoS One. 2016 May 26;11(5):e0156240. doi: 10.1371/journal.pone.0156240 (PMC4882018; doi:10.1371/journal.pone.0156240)
Supplement: S1 Table — (DOCX) [file pone.0156240.s002.docx]

**Supplemental Table 1. Associations of sociodemographic characteristics with friends’ past year alcohol use among high school youth^a^**

|  | **Total** | **Friends’ Past Year Alcohol Use** | | |  |
| --- | --- | --- | --- | --- | --- |
|  | **N (%)** | **No use^b^** | **Any use** | **--** | **p-value^e^** |
| **Full Sample Total N (%)** | 383 | 192 (50.1) | 191 (49.9) |  |  |
| **Age Groups** |  |  |  |  |  |
| ≤ 11 | 21 (5.5) | 21 (100) | 0 (0) |  | <.0001 |
| 12 - 15 | 136 (35.5) | 106 (77.9) | 30 (22.1) |  |  |
| 16 | 68 (17.8) | 32 (47.1) | 36 (52.9) |  |  |
| 17 | 77 (20.1) | 14 (18.2) | 63 (81.8) |  |  |
| 18 | 81 (21.1) | 19 (23.5) | 62 (76.5) |  |  |
| **Grade** |  |  |  |  |  |
| 3^rd^- 5^th^ | 10 (2.6) | 10 (100) | 0 (0) |  | <.0001 |
| 6^th^ - 8^th^ | 57 (14.9) | 53 (93.0) | 4 (7.0) |  |  |
| ≥ 9^th^ | 316 (82.5) | 129 (40.8) | 187 (59.2) |  |  |
|  | **N (%)** | **No use^b^** | **Drank but no binging^c^** | **Binge drank^d^** | **p-value^e^** |
| **High School Sample Total N (%)** | 316 | 129 (40.8) | 123 (38.9) | 64 (20.3) |  |
| **Sex** |  |  |  |  |  |
| Male | 148 (46.8) | 64 (43.2) | 55 (37.2) | 29 (19.6) | 0.7114 |
| Female | 168 (53.2) | 65 (38.7) | 68 (40.5) | 35 (20.8) |  |
| **Race/Ethnicity** |  |  |  |  |  |
| White, non-Hispanic | 235 (74.4) | 92 (39.1) | 93 (39.6) | 50 (21.3) | 0.8504 |
| Other | 72 (22.8) | 33 (45.8) | 27 (37.5) | 12 (16.7) |  |
| Missing | 9 (2.8) | 4 (44.4) | 3 (33.3) | 2 (22.2) |  |
| **Parent’s Education** |  |  |  |  |  |
| College graduate | 220 (69.6) | 88 (40.0) | 87 (39.5) | 45 (20.5) | 0.9157 |
| Non-college graduate | 80 (25.3) | 33 (41.3) | 30 (37.5) | 17 (21.3) |  |
| Don’t know/missing | 16 (5.1) | 8 (50.0) | 6 (37.5) | 2 (12.5) |  |
| **Chronic Condition** |  |  |  |  |  |
| Asthma & Cystic fibrosis | 78 (24.7) | 40 (51.3) | 25 (32.1) | 13 (16.7) | 0.4440 |
| Type 1 Diabetes | 78 (24.7) | 26 (33.3) | 33 (42.3) | 19 (24.4) |  |
| Inflammatory bowel disease | 81 (25.6) | 31 (38.3) | 33 (40.7) | 17 (21.0) |  |
| Juvenile idiopathic arthritis | 79 (25.0) | 32 (40.5) | 32 (40.5) | 15 (19.0) |  |

^a^ Data are presented as number (percentage) of participants; the ‘Total’ column provides column percentages while row percentages are included for AUD risk. Note that the ‘full sample’ includes all youth who reported on their friends’ drinking behaviors (N=383) while the ‘high school sample’ includes all high school aged youth who reported on their friends’ drinking behaviors (N=316).

^b^ No use: participants did not report friends’ use of alcohol in the past year

^c^ Drank but no binging: participants reported their friends drank in the past year, but did not report their friends binge drank in the past year

^d^ Binge drank: participants reported their friends binge drank in the past year

^e^ χ^2^ test for difference across categories.
