# Supplementary material for: A Screening Tool for Assessing Alcohol Use Risk among Medically Vulnerable Youth
Source: PLoS One. 2016 May 26;11(5):e0156240. doi: 10.1371/journal.pone.0156240 (PMC4882018; doi:10.1371/journal.pone.0156240)
Supplement: S2 Table — (DOCX) [file pone.0156240.s003.docx]

**Supplemental Table 2.Multivariate logistic regression predicting high school youth’s own risk for past year use of substance as predicted by friends’ past year use**

| **Outcome (own past year use)** | **Exposure (friends’ past year use)** | **OR (95% CI)^a^** |
| --- | --- | --- |
| Self-reported past year drinking  (vs. no drinking in past year) | Friends drank in past year | 12.60 (4.82 – 32.89) |
|  | Friends did not drink in past year | *1.00 (Reference)* |
|  | | |
| Self-report use of any substance  (vs. no use of alcohol/tobacco/ marijuana) | Friends binge drank in past year | 16.45 (5.35 – 50.64) |
|  | Friends drank but not binged in past year | 13.17 (5.73 – 30.29) |
|  | Friends did not drink in past year | *1.00 (Reference)* |
|  | | |
| Met criteria for a substance use disorder (vs. did not meet criteria for substance use disorder) | Friends binge drank in past year | 43.63 (6.63 – 286.98) |
|  | Friends drank but not binged in past year | 15.66 (2.72 – 90.03) |
|  | Friends did not drink in past year | *1.00 (Reference)* |

^a^ All estimates were obtained using generalized estimating equations (GEE) account for clustering within clinics and adjusting for age, sex, race/ethnicity, and parental education. Sample included all high school aged youth who reported on their friends’ drinking behaviors (N=316).
